# Ammonium tri-*tert*-butoxy­silanethiol­ate

**DOI:** 10.1107/S1600536808018370

**Published:** 2008-06-21

**Authors:** Katarzyna Baranowska, Ksymena Liadis, Wiesław Wojnowski

**Affiliations:** a Department of Inorganic Chemistry, Faculty of Chemistry, Gdańsk University of Technology, 11/12 G. Narutowicz Street, 80952 PL Gdańsk, Poland

## Abstract

The cations and anions of the title salt, NH_4_^+^·C_12_H_27_O_3_SSi^−^, are linked by N—H⋯S and N—H⋯O hydrogen bonds into a linear chain that runs along the *a* axis of the monoclinic unit cell. The asymmetric unit contains two cations and two anions.

## Related literature

For the synthesis of the thiol reagent, see: Piękoś & Wojnowski (1962 ▶). For an early study of its ammonium salt, see: Wojnowski (1971 ▶). For the structures of similar salts and comparison bond distances, see: Baranowska (2007 ▶); Baranowska, Chojnacki, Becker & Wojnowski (2003 ▶); Baranowska, Chojnacki, Gosiewska & Wojnowski (2006 ▶); Baranowska, Chojnacki, Konitz *et al.* (2006 ▶); Baranowska, Chojnacki, Wojnowski & Becker (2003 ▶); Becker *et al.* (2002 ▶, 2004 ▶); Chojnacki (2008 ▶); Dołęga *et al.* (2008 ▶); Pladzyk & Baranowska (2007 ▶). For the graph-set description of hydrogen-bonding patterns, see: Bernstein *et al.* (1995 ▶); Etter (1990 ▶).
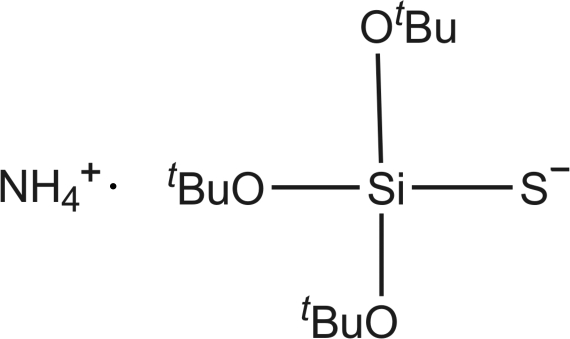


## Experimental

### 

#### Crystal data

NH_4_^+^·C_12_H_27_O_3_SSi^−^
*M* *_r_* = 297.53
Monoclinic, 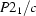

*a* = 11.9981 (4) Å
*b* = 12.5580 (5) Å
*c* = 24.8181 (12) Å
β = 100.336 (4)°
*V* = 3678.7 (3) Å^3^
*Z* = 8
Mo *K*α radiation
μ = 0.24 mm^−1^
*T* = 120 (2) K
0.2 × 0.06 × 0.04 mm

#### Data collection

Oxford Diffraction KM-4 CCD diffractometer
Absorption correction: none
12272 measured reflections
6433 independent reflections
4509 reflections with *I* > 2σ(*I*)
*R*_int_ = 0.028

#### Refinement

*R*[*F* ^2^ > 2σ(*F* ^2^)] = 0.048
*wR*(*F* ^2^) = 0.126
*S* = 1.04
6433 reflections
367 parameters
8 restraints
H atoms treated by a mixture of independent and constrained refinement
Δρ_max_ = 0.71 e Å^−3^
Δρ_min_ = −0.38 e Å^−3^

### 

Data collection: *CrysAlis CCD* (Oxford Diffraction, 2006 ▶); cell refinement: *CrysAlis RED* (Oxford Diffraction, 2006 ▶); data reduction: *CrysAlis RED*; program(s) used to solve structure: *SHELXS97* (Sheldrick, 2008 ▶); program(s) used to refine structure: *SHELXL97* (Sheldrick, 2008 ▶); molecular graphics: *ORTEP-3 for Windows* (Farrugia, 1997 ▶); software used to prepare material for publication: *WinGX* (Farrugia, 1999 ▶).

## Supplementary Material

Crystal structure: contains datablocks I, global. DOI: 10.1107/S1600536808018370/ng2465sup1.cif

Structure factors: contains datablocks I. DOI: 10.1107/S1600536808018370/ng2465Isup2.hkl

Additional supplementary materials:  crystallographic information; 3D view; checkCIF report

## Figures and Tables

**Table 1:** Hydrogen-bond geometry (Å, °)

*D*—H⋯*A*	*D*—H	H⋯*A*	*D*⋯*A*	*D*—H⋯*A*
N1—H1*A*⋯S1^i^	0.903 (17)	2.331 (18)	3.223 (2)	170 (2)
N1—H1*B*⋯S2	0.891 (17)	2.334 (18)	3.215 (2)	169 (3)
N1—H1*C*⋯S1	0.899 (18)	2.326 (19)	3.219 (3)	172 (3)
N1—H1*D*⋯O2	0.893 (17)	2.49 (2)	3.059 (3)	122 (2)
N2—H2*E*⋯S2^ii^	0.902 (19)	2.38 (2)	3.255 (3)	162 (4)
N2—H2*D*⋯O3	0.877 (19)	2.01 (2)	2.886 (3)	175 (4)
N2—H2*D*⋯S1	0.877 (19)	2.95 (4)	3.337 (3)	109 (3)
N2—H2*F*⋯S2	0.897 (18)	2.435 (19)	3.323 (3)	171 (3)
N2—H2*G*⋯O5^ii^	0.867 (18)	2.39 (3)	2.952 (3)	123 (3)

Symmetry codes: (i) 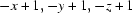; (ii) 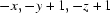.

**Fig. 1.**
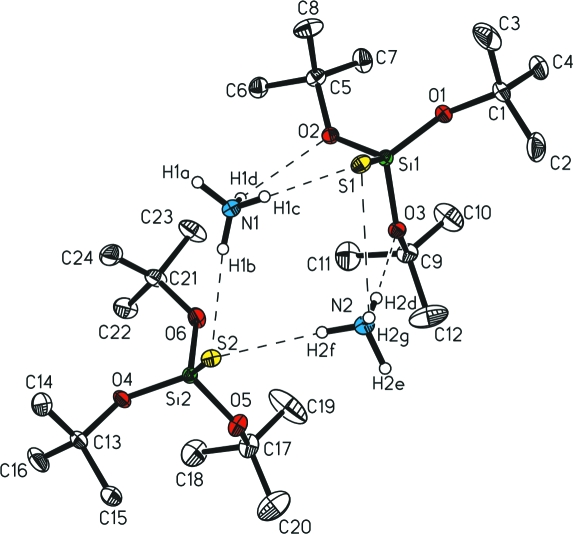
A view of the molecule of (I), showing the atom-labelling scheme. Displacement ellipsoids are drawn at the 30% probability level. All tBu H atoms have been omitted for clarity. Hydrogen bonds are indicated with dashed lines.

**Fig. 2.**
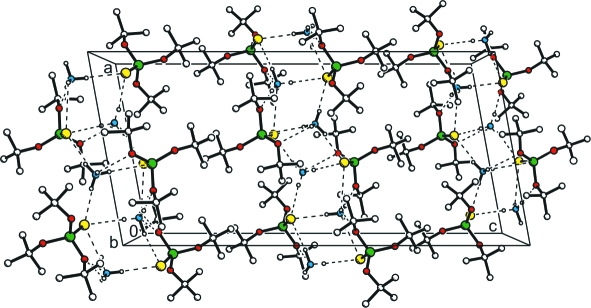
The chains of molecules of (I) linked by hydrogen bonds. A view direction is parallel to the crystallographic b axis. All tBu H atoms have been omitted for clarity. Hydrogen bonds are indicated with dashed lines.

## supplementary crystallographic information

### Comment

Complexes of trialkoxysilanethiols with ammonia were first obtained in 1971 by the reaction of (^i^PrO)_3_SiSH and (*^s^*BuO)_3_SiSH (Wojnowski, 1971) with ammonia but no structural data are available.

Amino tri-*tert*-butoxysilanethiolates show great structural diversity. Structures of isolated dimers, cubans, chains or sheets have been recognized so far. Cubic motifs were characterized for salts of primary amines (Becker, *et al.*, 2004); for secondary amines dimers and/or chains have been found (Baranowska, Chojnacki, Konitz *et al.*, 2006, Baranowska, Chojnacki, Becker & Wojnowski, 2003).

The asymmetric unit of (I) consists of two silanethiolate anions and two ammonium cations (Fig.1). The ions are connected *via* N^(+)^–H···S^(-)^ hydrogen bonds, forming an eight-membered ring as *R*^2^_4_(8) with four donors and two acceptors (Etter, 1990; Bernstein *et al.*, 1995). Similar (thiol-amine)_2_ ring formation has been observed in other ammonium salts (Baranowska, Chojnacki, Konitz *et al.*, 2006; Baranowska, Chojnacki, Wojnowski & Becker, 2003). These rings are futher linked by N–H···S and N–H···O hydrogen bonds into chains parallel to the (010) (Fig.2). The application of graph theory to the chain results in a variety of possible hydrogen bonding patterns: the N1–H1C···S1, N1–H1A···S1^(i)^, N1–H1C···S1^(i)^ and N1–H1A···S1 hydrogen bonds form an *R*^2^_4_(8)ring, the N2–H2E···S2^(ii)^, N2–H2F···S2^(ii)^, N2–H2F···S2 and N2–H2E···S2 hydrogen bonds form an *R*^2^_4_(8) ring, the N1–H1C···S1and N1–H1D···O2 hydrogen bonds form an *R*^2^_2_(6) ring, the N2–H2G···O5^(ii)^and N2–H2E···S2^(ii)^ hydrogen bonds form an *R*^1^_2_(6) ring, the N1–H1B···S2, N2–H2F···S2, N1–H1C···S1 and N2–H2D···S1 hydrogen bonds form an *R*^2^_4_(8) ring, in which atom N2 acts as a bifurcated donor.

The N···S distances in (I) lie in the range 2.886 (3)–3.340 (3) Å, comparable with values observed in other silanethiolates (Baranowska, Chojnacki, Gosiewska & Wojnowski 2006; Pladzyk & Baranowska, 2007; Dołęga *et al.*, 2008) or aromatic thiolates (Baranowska, Chojnacki, Becker & Wojnowski, 2003; Baranowska, 2007). The length of silicon – sulfur bond is short (2.059 – 2.062 Å) and it is characteristic for ionic silanethiolates (Becker *et al.*, 2002, Chojnacki, 2008). Hydrogen bonds are grouped in " hydrophilic" core, while the organic hydrocarbon groups form "hydrophobic" coat in the crystal. It is a very characteristic motif for many polimeric structures.

### Experimental

The substrate (*^t^*BuO)_3_SiSH was prepared according to the literature (Piękoś & Wojnowski, 1962).

To the mixture of to tri-*tert*-butoxysilanethiol (1.9 g; 0.0064 mol*)* and metallic magnesium (0.084 g; 0.0035 mol) in toluene one drop of concentrated ammonia solution was added. The mixture was stirred and heated at boiling point of toluene for one week. The solution was separated from the metal by filtration. The solvent was removed. The liquid residue was kept in refrigerator (4 °C) for a few days for crystallization. The final product has a form of colourless, crystalline needles.

### Refinement

All H atoms were refined as riding on C atoms with methyl C—H = 0.98 Å, and 1.5*U*_eq_(C) for CH_3_ groups. Hydrogen atoms of ammonium were found in the electron density Fourier map and were refined with N—H bond lengths constrained to 0.89 (2) Å.

### Crystal data

**Table:**

NH_4_^+^·C_12_H_27_O_3_SSi^–^	*F*_000_ = 1312
*M**_r_* = 297.53	*D*_x_ = 1.074 Mg m^−^^3^
Monoclinic, *P*2_1_/*c*	Mo *K*α radiation λ = 0.71073 Å
Hall symbol: -P 2ybc	Cell parameters from 7143 reflections
*a* = 11.9981 (4) Å	θ = 2.0–32.4º
*b* = 12.5580 (5) Å	µ = 0.24 mm^−^^1^
*c* = 24.8181 (12) Å	*T* = 120 (2) K
β = 100.336 (4)º	Prism, colourless
*V* = 3678.7 (3) Å^3^	0.2 × 0.06 × 0.04 mm
*Z* = 8	

### Data collection

**Table:**

Oxford Diffraction KM-4 CCD diffractometer	4509 reflections with *I* > 2σ(*I*)
Monochromator: graphite	*R*_int_ = 0.028
Detector resolution: 8.1883 pixels mm^-1^	θ_max_ = 25.1º
*T* = 120(2) K	θ_min_ = 2.4º
0.75° width ω scans	*h* = −14→8
Absorption correction: none	*k* = −14→8
12272 measured reflections	*l* = −29→29
6433 independent reflections	

### Refinement

**Table:**

Refinement on *F*^2^	Secondary atom site location: difference Fourier map
Least-squares matrix: full	Hydrogen site location: inferred from neighbouring sites
*R*[*F*^2^ > 2σ(*F*^2^)] = 0.048	H atoms treated by a mixture of independent and constrained refinement
*wR*(*F*^2^) = 0.126	*w* = 1/[σ^2^(*F*_o_^2^) + (0.0746*P*)^2^] where *P* = (*F*_o_^2^ + 2*F*_c_^2^)/3
*S* = 1.04	(Δ/σ)_max_ = 0.001
6433 reflections	Δρ_max_ = 0.72 e Å^−^^3^
367 parameters	Δρ_min_ = −0.38 e Å^−^^3^
8 restraints	Extinction correction: none
Primary atom site location: structure-invariant direct methods	

### Special details

**Table:**

Geometry. All e.s.d.'s (except the e.s.d. in the dihedral angle between two l.s. planes) are estimated using the full covariance matrix. The cell e.s.d.'s are taken into account individually in the estimation of e.s.d.'s in distances, angles and torsion angles; correlations between e.s.d.'s in cell parameters are only used when they are defined by crystal symmetry. An approximate (isotropic) treatment of cell e.s.d.'s is used for estimating e.s.d.'s involving l.s. planes.
Refinement. Refinement of *F*^2^ against ALL reflections. The weighted *R*-factor *wR* and goodness of fit *S* are based on *F*^2^, conventional *R*-factors *R* are based on *F*, with *F* set to zero for negative *F*^2^. The threshold expression of *F*^2^ > σ(*F*^2^) is used only for calculating *R*-factors(gt) *etc*. and is not relevant to the choice of reflections for refinement. *R*-factors based on *F*^2^ are statistically about twice as large as those based on *F*, and *R*- factors based on ALL data will be even larger.

### Fractional atomic coordinates and isotropic or equivalent isotropic displacement parameters (Å^2^)

**Table:**

	*x*	*y*	*z*	*U*_iso_*/*U*_eq_	
S1	0.42331 (5)	0.53353 (5)	0.58655 (3)	0.02431 (17)	
Si1	0.42375 (6)	0.37946 (6)	0.61491 (3)	0.01975 (18)	
O1	0.49073 (14)	0.35757 (14)	0.67658 (7)	0.0264 (4)	
O2	0.47413 (14)	0.29078 (13)	0.57685 (7)	0.0236 (4)	
O3	0.28781 (14)	0.34803 (14)	0.60815 (7)	0.0234 (4)	
C1	0.5191 (2)	0.4195 (3)	0.72592 (12)	0.0377 (7)	
C2	0.4156 (3)	0.4800 (3)	0.73596 (13)	0.0554 (10)	
H2A	0.3533	0.43	0.7369	0.083*	
H2B	0.3932	0.5318	0.7064	0.083*	
H2C	0.4334	0.5176	0.7711	0.083*	
C3	0.6160 (3)	0.4934 (4)	0.72026 (16)	0.0703 (12)	
H3A	0.5911	0.5443	0.6906	0.105*	
H3B	0.6797	0.4516	0.7118	0.105*	
H3C	0.64	0.5321	0.7547	0.105*	
C4	0.5552 (3)	0.3381 (3)	0.77117 (12)	0.0550 (10)	
H4A	0.4924	0.2889	0.7727	0.082*	
H4B	0.5757	0.3749	0.8064	0.082*	
H4C	0.6207	0.298	0.7635	0.082*	
C5	0.5858 (2)	0.2435 (2)	0.58098 (11)	0.0273 (6)	
C6	0.5803 (2)	0.1793 (2)	0.52880 (12)	0.0351 (7)	
H6A	0.5641	0.2269	0.4971	0.053*	
H6B	0.5202	0.1258	0.5265	0.053*	
H6C	0.6531	0.1437	0.5291	0.053*	
C7	0.6098 (3)	0.1701 (3)	0.63015 (12)	0.0424 (8)	
H7A	0.6108	0.2115	0.6637	0.064*	
H7B	0.6835	0.1357	0.6314	0.064*	
H7C	0.5505	0.1156	0.6272	0.064*	
C8	0.6743 (2)	0.3297 (2)	0.58404 (15)	0.0450 (9)	
H8A	0.6592	0.3729	0.5506	0.067*	
H8B	0.7495	0.2972	0.5876	0.067*	
H8C	0.6715	0.3751	0.6159	0.067*	
C9	0.2329 (2)	0.2472 (2)	0.61567 (12)	0.0317 (7)	
C10	0.2878 (3)	0.1914 (3)	0.66557 (15)	0.0667 (12)	
H10A	0.3677	0.1788	0.6638	0.1*	
H10B	0.2497	0.1232	0.6684	0.1*	
H10C	0.2825	0.2352	0.6977	0.1*	
C11	0.2340 (3)	0.1800 (3)	0.56391 (15)	0.0600 (10)	
H11A	0.2038	0.2223	0.5314	0.09*	
H11B	0.187	0.1165	0.5649	0.09*	
H11C	0.3119	0.1585	0.5625	0.09*	
C12	0.1101 (3)	0.2741 (3)	0.6167 (2)	0.0734 (13)	
H12A	0.0763	0.3086	0.5822	0.11*	
H12B	0.1061	0.3224	0.6473	0.11*	
H12C	0.0684	0.2086	0.6213	0.11*	
S2	0.10838 (5)	0.46097 (5)	0.41794 (3)	0.02520 (18)	
Si2	0.05808 (6)	0.31006 (6)	0.39125 (3)	0.02122 (18)	
O4	0.02593 (15)	0.29638 (15)	0.32505 (7)	0.0286 (4)	
O5	−0.05329 (15)	0.28237 (14)	0.41883 (8)	0.0311 (5)	
O6	0.15294 (16)	0.21879 (15)	0.41046 (7)	0.0348 (5)	
C13	−0.0289 (2)	0.3627 (3)	0.28096 (11)	0.0349 (7)	
C14	0.0582 (3)	0.4383 (3)	0.26532 (14)	0.0525 (10)	
H14A	0.0894	0.4827	0.2969	0.079*	
H14B	0.0223	0.4838	0.2351	0.079*	
H14C	0.1194	0.3973	0.2538	0.079*	
C15	−0.1263 (3)	0.4238 (3)	0.29753 (13)	0.0468 (9)	
H15A	−0.0971	0.4715	0.3281	0.07*	
H15B	−0.1803	0.3737	0.3088	0.07*	
H15C	−0.1645	0.4659	0.2664	0.07*	
C16	−0.0726 (3)	0.2868 (3)	0.23444 (13)	0.0578 (10)	
H16A	−0.1298	0.2397	0.2453	0.087*	
H16B	−0.0096	0.244	0.226	0.087*	
H16C	−0.1065	0.3276	0.202	0.087*	
C17	−0.1164 (3)	0.1842 (2)	0.42078 (12)	0.0354 (7)	
C18	−0.1311 (3)	0.1229 (3)	0.36650 (16)	0.0608 (10)	
H18A	−0.1662	0.1694	0.3365	0.091*	
H18B	−0.1796	0.0607	0.3684	0.091*	
H18C	−0.0568	0.0993	0.3599	0.091*	
C19	−0.0602 (4)	0.1198 (3)	0.4673 (2)	0.1000 (19)	
H19A	0.0185	0.1064	0.4636	0.15*	
H19B	−0.1002	0.0518	0.4678	0.15*	
H19C	−0.0616	0.1582	0.5016	0.15*	
C20	−0.2341 (3)	0.2171 (3)	0.4273 (2)	0.0743 (13)	
H20A	−0.2662	0.2647	0.3972	0.111*	
H20B	−0.2305	0.2544	0.4623	0.111*	
H20C	−0.2819	0.1538	0.4267	0.111*	
C21	0.2338 (2)	0.1648 (2)	0.38299 (12)	0.0357 (7)	
C22	0.1726 (3)	0.0832 (3)	0.34325 (14)	0.0484 (9)	
H22A	0.1192	0.1196	0.3146	0.073*	
H22B	0.1311	0.0338	0.363	0.073*	
H22C	0.2279	0.0436	0.3265	0.073*	
C23	0.3137 (3)	0.1104 (3)	0.42870 (14)	0.0545 (10)	
H23A	0.3499	0.164	0.4548	0.082*	
H23B	0.3719	0.0719	0.4134	0.082*	
H23C	0.2712	0.0602	0.4475	0.082*	
C24	0.2952 (3)	0.2437 (3)	0.35263 (13)	0.0482 (9)	
H24A	0.2411	0.2768	0.3231	0.072*	
H24B	0.3539	0.2065	0.337	0.072*	
H24C	0.3305	0.2988	0.3781	0.072*	
N1	0.36606 (19)	0.40699 (19)	0.47213 (10)	0.0245 (5)	
H1A	0.419 (2)	0.423 (2)	0.4515 (10)	0.038*	
H1B	0.2948 (16)	0.414 (2)	0.4546 (11)	0.038*	
H1C	0.380 (3)	0.449 (2)	0.5020 (10)	0.048*	
H1D	0.379 (2)	0.3393 (15)	0.4824 (11)	0.026*	
N2	0.1426 (2)	0.5109 (2)	0.55138 (11)	0.0333 (6)	
H2E	0.071 (2)	0.503 (4)	0.5577 (19)	0.113*	
H2D	0.185 (3)	0.463 (3)	0.5707 (16)	0.09*	
H2F	0.137 (3)	0.490 (3)	0.5165 (8)	0.047*	
H2G	0.166 (3)	0.5762 (17)	0.5557 (15)	0.065*	

### Atomic displacement parameters (Å^2^)

**Table:**

	*U*^11^	*U*^22^	*U*^33^	*U*^12^	*U*^13^	*U*^23^
S1	0.0224 (3)	0.0208 (3)	0.0313 (4)	−0.0013 (3)	0.0089 (3)	0.0004 (3)
Si1	0.0197 (4)	0.0211 (4)	0.0188 (4)	−0.0014 (3)	0.0044 (3)	0.0005 (3)
O1	0.0250 (9)	0.0321 (10)	0.0209 (10)	0.0016 (8)	0.0012 (8)	−0.0008 (8)
O2	0.0231 (9)	0.0238 (10)	0.0237 (10)	0.0039 (8)	0.0038 (8)	0.0002 (8)
O3	0.0214 (9)	0.0242 (10)	0.0249 (10)	−0.0036 (8)	0.0047 (8)	0.0026 (8)
C1	0.0325 (16)	0.055 (2)	0.0232 (15)	−0.0012 (15)	−0.0011 (13)	−0.0124 (15)
C2	0.066 (2)	0.069 (3)	0.0322 (18)	0.020 (2)	0.0104 (17)	−0.0167 (18)
C3	0.063 (2)	0.088 (3)	0.053 (2)	−0.036 (2)	−0.0059 (19)	−0.026 (2)
C4	0.047 (2)	0.091 (3)	0.0240 (17)	0.013 (2)	−0.0014 (15)	−0.0020 (18)
C5	0.0262 (14)	0.0211 (14)	0.0352 (16)	0.0072 (12)	0.0071 (12)	0.0045 (13)
C6	0.0375 (16)	0.0343 (17)	0.0347 (17)	0.0114 (14)	0.0097 (14)	0.0008 (14)
C7	0.054 (2)	0.0396 (19)	0.0324 (17)	0.0204 (16)	0.0039 (15)	0.0047 (15)
C8	0.0272 (16)	0.0332 (18)	0.076 (3)	0.0023 (14)	0.0138 (16)	−0.0061 (17)
C9	0.0296 (15)	0.0264 (16)	0.0399 (17)	−0.0114 (13)	0.0086 (13)	0.0069 (14)
C10	0.073 (3)	0.063 (2)	0.057 (2)	−0.033 (2)	−0.008 (2)	0.030 (2)
C11	0.072 (3)	0.049 (2)	0.060 (2)	−0.028 (2)	0.015 (2)	−0.0103 (19)
C12	0.0363 (19)	0.051 (2)	0.137 (4)	−0.0139 (18)	0.027 (2)	0.021 (3)
S2	0.0219 (3)	0.0236 (4)	0.0301 (4)	0.0002 (3)	0.0046 (3)	−0.0017 (3)
Si2	0.0227 (4)	0.0215 (4)	0.0199 (4)	0.0040 (3)	0.0049 (3)	−0.0008 (3)
O4	0.0289 (10)	0.0335 (11)	0.0208 (10)	0.0077 (9)	−0.0026 (8)	−0.0002 (9)
O5	0.0376 (11)	0.0216 (10)	0.0378 (12)	−0.0063 (9)	0.0168 (9)	−0.0036 (9)
O6	0.0428 (12)	0.0355 (12)	0.0250 (10)	0.0195 (10)	0.0032 (9)	−0.0034 (9)
C13	0.0307 (15)	0.0506 (19)	0.0217 (15)	0.0130 (14)	0.0006 (12)	0.0056 (14)
C14	0.0428 (19)	0.077 (3)	0.0389 (19)	0.0078 (18)	0.0116 (16)	0.0254 (19)
C15	0.0415 (18)	0.064 (2)	0.0351 (18)	0.0239 (17)	0.0062 (15)	0.0184 (17)
C16	0.059 (2)	0.083 (3)	0.0257 (17)	0.013 (2)	−0.0085 (16)	−0.0033 (18)
C17	0.0470 (18)	0.0211 (15)	0.0411 (18)	−0.0124 (13)	0.0158 (15)	−0.0007 (13)
C18	0.060 (2)	0.055 (2)	0.069 (3)	−0.0186 (19)	0.017 (2)	−0.021 (2)
C19	0.119 (4)	0.060 (3)	0.095 (4)	−0.049 (3)	−0.050 (3)	0.049 (3)
C20	0.064 (2)	0.051 (2)	0.119 (4)	−0.020 (2)	0.047 (3)	−0.004 (2)
C21	0.0326 (16)	0.0345 (17)	0.0359 (17)	0.0169 (14)	−0.0052 (13)	−0.0168 (14)
C22	0.0457 (19)	0.047 (2)	0.049 (2)	0.0105 (16)	−0.0014 (16)	−0.0230 (17)
C23	0.056 (2)	0.042 (2)	0.055 (2)	0.0265 (17)	−0.0198 (17)	−0.0226 (17)
C24	0.0380 (18)	0.062 (2)	0.046 (2)	0.0076 (17)	0.0101 (15)	−0.0189 (18)
N1	0.0226 (12)	0.0240 (12)	0.0279 (13)	0.0002 (11)	0.0069 (10)	0.0001 (11)
N2	0.0261 (13)	0.0430 (16)	0.0310 (14)	0.0058 (12)	0.0058 (11)	−0.0021 (13)

### Geometric parameters (Å, °)

**Table:**

S1—Si1	2.0586 (10)	O4—C13	1.437 (3)
Si1—O1	1.6194 (18)	O5—C17	1.452 (3)
Si1—O2	1.6439 (18)	O6—C21	1.450 (3)
Si1—O3	1.6570 (17)	C13—C14	1.514 (4)
O1—C1	1.439 (3)	C13—C15	1.515 (4)
O2—C5	1.452 (3)	C13—C16	1.516 (4)
O3—C9	1.454 (3)	C14—H14A	0.98
C1—C3	1.514 (5)	C14—H14B	0.98
C1—C2	1.514 (4)	C14—H14C	0.98
C1—C4	1.523 (5)	C15—H15A	0.98
C2—H2A	0.98	C15—H15B	0.98
C2—H2B	0.98	C15—H15C	0.98
C2—H2C	0.98	C16—H16A	0.98
C3—H3A	0.98	C16—H16B	0.98
C3—H3B	0.98	C16—H16C	0.98
C3—H3C	0.98	C17—C19	1.471 (5)
C4—H4A	0.98	C17—C20	1.507 (4)
C4—H4B	0.98	C17—C18	1.534 (4)
C4—H4C	0.98	C18—H18A	0.98
C5—C8	1.509 (4)	C18—H18B	0.98
C5—C7	1.515 (4)	C18—H18C	0.98
C5—C6	1.517 (4)	C19—H19A	0.98
C6—H6A	0.98	C19—H19B	0.98
C6—H6B	0.98	C19—H19C	0.98
C6—H6C	0.98	C20—H20A	0.98
C7—H7A	0.98	C20—H20B	0.98
C7—H7B	0.98	C20—H20C	0.98
C7—H7C	0.98	C21—C23	1.511 (4)
C8—H8A	0.98	C21—C24	1.514 (5)
C8—H8B	0.98	C21—C22	1.517 (4)
C8—H8C	0.98	C22—H22A	0.98
C9—C10	1.472 (4)	C22—H22B	0.98
C9—C12	1.517 (4)	C22—H22C	0.98
C9—C11	1.539 (4)	C23—H23A	0.98
C10—H10A	0.98	C23—H23B	0.98
C10—H10B	0.98	C23—H23C	0.98
C10—H10C	0.98	C24—H24A	0.98
C11—H11A	0.98	C24—H24B	0.98
C11—H11B	0.98	C24—H24C	0.98
C11—H11C	0.98	N1—H1A	0.903 (17)
C12—H12A	0.98	N1—H1B	0.891 (17)
C12—H12B	0.98	N1—H1C	0.899 (18)
C12—H12C	0.98	N1—H1D	0.893 (17)
S2—Si2	2.0619 (10)	N2—H2E	0.902 (19)
Si2—O6	1.6253 (19)	N2—H2D	0.877 (19)
Si2—O4	1.6279 (19)	N2—H2F	0.897 (18)
Si2—O5	1.6440 (18)	N2—H2G	0.867 (18)
			
O1—Si1—O2	104.88 (9)	C13—O4—Si2	134.46 (17)
O1—Si1—O3	111.58 (9)	C17—O5—Si2	131.38 (17)
O2—Si1—O3	103.72 (9)	C21—O6—Si2	133.17 (18)
O1—Si1—S1	116.94 (8)	O4—C13—C14	108.4 (2)
O2—Si1—S1	114.76 (7)	O4—C13—C15	110.9 (2)
O3—Si1—S1	104.31 (7)	C14—C13—C15	110.7 (3)
C1—O1—Si1	135.56 (18)	O4—C13—C16	105.3 (3)
C5—O2—Si1	131.79 (16)	C14—C13—C16	111.0 (3)
C9—O3—Si1	130.88 (16)	C15—C13—C16	110.3 (3)
O1—C1—C3	108.7 (3)	C13—C14—H14A	109.5
O1—C1—C2	109.6 (2)	C13—C14—H14B	109.5
C3—C1—C2	112.0 (3)	H14A—C14—H14B	109.5
O1—C1—C4	104.9 (3)	C13—C14—H14C	109.5
C3—C1—C4	111.0 (3)	H14A—C14—H14C	109.5
C2—C1—C4	110.4 (3)	H14B—C14—H14C	109.5
C1—C2—H2A	109.5	C13—C15—H15A	109.5
C1—C2—H2B	109.5	C13—C15—H15B	109.5
H2A—C2—H2B	109.5	H15A—C15—H15B	109.5
C1—C2—H2C	109.5	C13—C15—H15C	109.5
H2A—C2—H2C	109.5	H15A—C15—H15C	109.5
H2B—C2—H2C	109.5	H15B—C15—H15C	109.5
C1—C3—H3A	109.5	C13—C16—H16A	109.5
C1—C3—H3B	109.5	C13—C16—H16B	109.5
H3A—C3—H3B	109.5	H16A—C16—H16B	109.5
C1—C3—H3C	109.5	C13—C16—H16C	109.5
H3A—C3—H3C	109.5	H16A—C16—H16C	109.5
H3B—C3—H3C	109.5	H16B—C16—H16C	109.5
C1—C4—H4A	109.5	O5—C17—C19	109.0 (3)
C1—C4—H4B	109.5	O5—C17—C20	106.0 (2)
H4A—C4—H4B	109.5	C19—C17—C20	111.8 (4)
C1—C4—H4C	109.5	O5—C17—C18	112.1 (2)
H4A—C4—H4C	109.5	C19—C17—C18	112.2 (3)
H4B—C4—H4C	109.5	C20—C17—C18	105.7 (3)
O2—C5—C8	110.0 (2)	C17—C18—H18A	109.5
O2—C5—C7	110.2 (2)	C17—C18—H18B	109.5
C8—C5—C7	111.5 (3)	H18A—C18—H18B	109.5
O2—C5—C6	105.0 (2)	C17—C18—H18C	109.5
C8—C5—C6	110.3 (2)	H18A—C18—H18C	109.5
C7—C5—C6	109.7 (2)	H18B—C18—H18C	109.5
C5—C6—H6A	109.5	C17—C19—H19A	109.5
C5—C6—H6B	109.5	C17—C19—H19B	109.5
H6A—C6—H6B	109.5	H19A—C19—H19B	109.5
C5—C6—H6C	109.5	C17—C19—H19C	109.5
H6A—C6—H6C	109.5	H19A—C19—H19C	109.5
H6B—C6—H6C	109.5	H19B—C19—H19C	109.5
C5—C7—H7A	109.5	C17—C20—H20A	109.5
C5—C7—H7B	109.5	C17—C20—H20B	109.5
H7A—C7—H7B	109.5	H20A—C20—H20B	109.5
C5—C7—H7C	109.5	C17—C20—H20C	109.5
H7A—C7—H7C	109.5	H20A—C20—H20C	109.5
H7B—C7—H7C	109.5	H20B—C20—H20C	109.5
C5—C8—H8A	109.5	O6—C21—C23	104.3 (2)
C5—C8—H8B	109.5	O6—C21—C24	110.7 (2)
H8A—C8—H8B	109.5	C23—C21—C24	111.6 (3)
C5—C8—H8C	109.5	O6—C21—C22	109.6 (2)
H8A—C8—H8C	109.5	C23—C21—C22	110.6 (3)
H8B—C8—H8C	109.5	C24—C21—C22	109.9 (3)
O3—C9—C10	112.2 (2)	C21—C22—H22A	109.5
O3—C9—C12	105.8 (2)	C21—C22—H22B	109.5
C10—C9—C12	112.4 (3)	H22A—C22—H22B	109.5
O3—C9—C11	107.4 (2)	C21—C22—H22C	109.5
C10—C9—C11	111.8 (3)	H22A—C22—H22C	109.5
C12—C9—C11	106.9 (3)	H22B—C22—H22C	109.5
C9—C10—H10A	109.5	C21—C23—H23A	109.5
C9—C10—H10B	109.5	C21—C23—H23B	109.5
H10A—C10—H10B	109.5	H23A—C23—H23B	109.5
C9—C10—H10C	109.5	C21—C23—H23C	109.5
H10A—C10—H10C	109.5	H23A—C23—H23C	109.5
H10B—C10—H10C	109.5	H23B—C23—H23C	109.5
C9—C11—H11A	109.5	C21—C24—H24A	109.5
C9—C11—H11B	109.5	C21—C24—H24B	109.5
H11A—C11—H11B	109.5	H24A—C24—H24B	109.5
C9—C11—H11C	109.5	C21—C24—H24C	109.5
H11A—C11—H11C	109.5	H24A—C24—H24C	109.5
H11B—C11—H11C	109.5	H24B—C24—H24C	109.5
C9—C12—H12A	109.5	H1A—N1—H1B	114 (3)
C9—C12—H12B	109.5	H1A—N1—H1C	107 (3)
H12A—C12—H12B	109.5	H1B—N1—H1C	112 (3)
C9—C12—H12C	109.5	H1A—N1—H1D	106 (2)
H12A—C12—H12C	109.5	H1B—N1—H1D	110 (3)
H12B—C12—H12C	109.5	H1C—N1—H1D	109 (3)
O6—Si2—O4	104.38 (10)	H2E—N2—H2D	108 (4)
O6—Si2—O5	107.90 (11)	H2E—N2—H2F	103 (4)
O4—Si2—O5	109.56 (10)	H2D—N2—H2F	105 (3)
O6—Si2—S2	113.87 (8)	H2E—N2—H2G	113 (4)
O4—Si2—S2	115.08 (8)	H2D—N2—H2G	116 (4)
O5—Si2—S2	105.88 (7)	H2F—N2—H2G	111 (3)

### Hydrogen-bond geometry (Å, °)

**Table:**

*D*—H···*A*	*D*—H	H···*A*	*D*···*A*	*D*—H···*A*
N1—H1A···S1^i^	0.903 (17)	2.331 (18)	3.223 (2)	170 (2)
N1—H1B···S2	0.891 (17)	2.334 (18)	3.215 (2)	169 (3)
N1—H1C···S1	0.899 (18)	2.326 (19)	3.219 (3)	172 (3)
N1—H1D···O2	0.893 (17)	2.49 (2)	3.059 (3)	122 (2)
N2—H2E···S2^ii^	0.902 (19)	2.38 (2)	3.255 (3)	162 (4)
N2—H2D···O3	0.877 (19)	2.01 (2)	2.886 (3)	175 (4)
N2—H2D···S1	0.877 (19)	2.95 (4)	3.337 (3)	109 (3)
N2—H2F···S2	0.897 (18)	2.435 (19)	3.323 (3)	171 (3)
N2—H2G···O5^ii^	0.867 (18)	2.39 (3)	2.952 (3)	123 (3)

Symmetry codes: (i) −*x*+1, −*y*+1, −*z*+1; (ii) −*x*, −*y*+1, −*z*+1.
